# Simple liver cysts and cystoid lesions in hepatic alveolar echinococcosis: a retrospective cohort study with Hounsfield analysis

**DOI:** 10.1051/parasite/2019057

**Published:** 2019-08-30

**Authors:** Agata Engler, Rong Shi, Meinrad Beer, Julian Schmidberger, Wolfgang Kratzer, Thomas F. E. Barth, Johannes Grimm, Andreas Hillenbrand, Doris Henne-Bruns, Beate Gruener, Ambros J. Beer, Tilmann Graeter

**Affiliations:** 1 Department of Diagnostic and Interventional Radiology, University Hospital Ulm Albert-Einstein-Allee 23 89081 Ulm Germany; 2 Department of Internal Medicine I, University Hospital Ulm Albert-Einstein-Allee 23 89081 Ulm Germany; 3 Department of Pathology, University Hospital Ulm Albert-Einstein-Allee 23 89081 Ulm Germany; 4 Department of General and Visceral Surgery, University Hospital Ulm Albert-Einstein-Allee 23 89081 Ulm Germany; 5 Department of Internal Medicine III, University Hospital Ulm Albert-Einstein-Allee 23 89081 Ulm Germany; 6 Department of Nuclear Medicine, Ulm University Hospital Albert-Einstein-Allee 23 89081 Ulm Germany

**Keywords:** Alveolar echinococcosis, *Echinococcus multilocularis*, Hepatic cysts, Density, Computed tomography, Classification

## Abstract

**Background**. Alveolar echinococcosis (AE) is a rare zoonosis caused by the larval stage of the tapeworm *Echinococcus multilocularis*. AE lesions affect the liver in more than 98% of cases. AE lesions have various morphological characteristics that are described in the *Echinococcus multilocularis* Ulm classification for computed tomography (EMUC-CT). One of these characteristics is a cystoid portion. The aim of the study was to compare the density of simple hepatic cysts with cystoid portions of AE lesions classified on the basis of the EMUC-CT. **Results**. Hounsfield Unit (HU) measurements of the cystoid portions of all EMUC-CT type I–IV AE lesions (*n* = 155) gave a mean of 21.8 ± 17.6, which was significantly different from that of 2.9 ± 4.5 for the simple hepatic cysts (*p* < 0.0001). The difference between each of the individual AE types and simple hepatic cysts was also significant. In addition, the HU values of the cystoid portions in types I, II and IIIa/b and simple cysts were each significantly different from type IV (*p* < 0.0001). The HU measurements in type IV presented by far the highest mean. **Conclusions.** The significantly higher density measured in the cystoid portions of hepatic AE lesions offers a good means of differentiation from simple hepatic cysts.

## Abstract (fr)

**Contexte.** L’échinococcose alvéolaire (EA) est une zoonose rare causée par le stade larvaire du cestode *Echinococcus multilocularis*. Les lésions d’EA affectent le foie dans plus de 98 % des cas. Les lésions d’EA ont différentes caractéristiques morphologiques décrites dans la classification d’Ulm pour la tomodensitométrie d’*Echinococcus multilocularis* (EMUC-CT). Une de ces caractéristiques est une portion cystoïde. Le but de l’étude était de comparer la densité de kystes hépatiques simples avec des portions cystoïdes de lésions d’AE classées sur la base du EMUC-CT. **Résultats.** La mesure en UH (Unités Hounsfield) des portions cystoïdes de toutes les lésions d’EA de type I–IV EMUC-CT (*n* = 155) a donné une moyenne de 21,8 ± 17,6, significativement différente de celle de 2,9 ± 4,5 pour les kystes hépatiques simples (*p* < 0,0001). La différence entre chacun des types d’EA individuels et les kystes hépatiques simples était également significative. De plus, les valeurs d’UH des portions de cystoïde des types I, II et IIIa/b et des kystes simples étaient chacune significativement différentes de celles du type IV (*p* < 0,0001). Les mesures d’UH pour le type IV présentaient de loin la moyenne la plus élevée. **Conclusions.** La densité significativement plus élevée mesurée dans les parties cystoïdes des lésions d’EA hépatiques offre un bon moyen de différenciation par rapport aux kystes hépatiques simples.

## Introduction

Alveolar echinococcosis (AE) is a rare zoonosis caused by the larval stage of the fox tapeworm (*Echinococcus multilocularis*). Humans may become infected by ingesting contaminated food or direct contact with animals [1, 37]. In such cases, humans act as aberrant intermediate hosts, as they do not belong to the natural life cycle of the parasite [10] and the formation of protoscolices is possible only in exceptional cases [8]. The liver is the organ most commonly affected [1], and the infiltrative growth of the lesions is similar to that of a malignant tumour [18, 33, 34]. The disease is the most dangerous parasitic zoonosis in Europe. Left untreated, it has a very high mortality rate [1, 10]. One therapeutic option in terms of curative treatment is complete resection of the lesion, but this depends on the stage of the disease. Surgical excision may therefore contribute to cure [6]. If surgery is no longer an option, long-term pharmacotherapy with benzimidazole (BMZ) derivatives is indicated. Such treatment may inhibit further growth of the lesion and achieve a stable phase of the disease [4, 5, 10, 31]. Curative treatment of AE is only possible with early diagnosis [6, 9]. WHO and the European Echinococcosis Registry developed a classification similar to the TNM (T = Primary tumour, N = lymph nodes, M = metastases) system for malignant tumours to allow for better assessment of the disease stage. Diagnostic imaging forms the basis of the so-called PNM (P = parasitic mass in the liver, N = involvement of neighbouring organs, M = metastasis) classification. The classification allows for standardised staging upon initial diagnosis, which ensures optimal treatment [20]. Besides the case history, the diagnosis of AE requires a combination of imaging techniques such as ultrasound (US), computed tomography (CT), magnetic resonance imaging (MRI), and 18F fluorodeoxyglucose positron emission tomography/computed tomography (18F-FDG PET/CT), and the results of immunodiagnostics (specific serology). Histopathological findings and molecular DNA identification are used to confirm the diagnosis [39].

**Figure 1:**
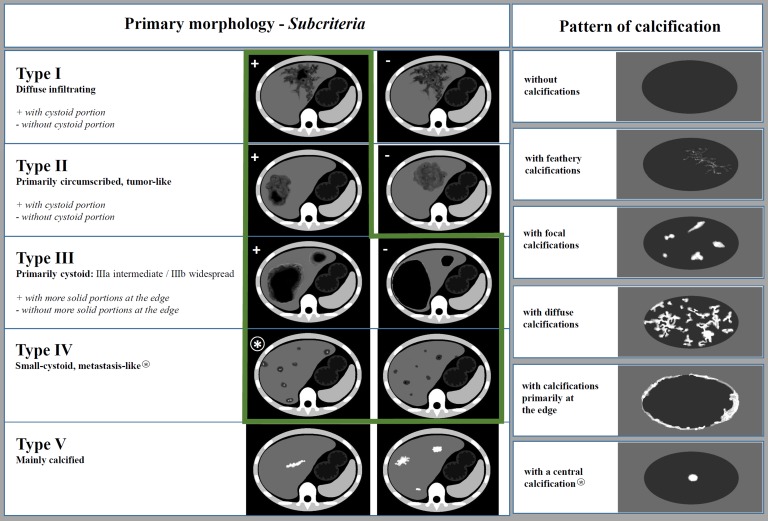
Overview of the EMUC-CT after Graeter et al. Left: Primary morphological types I–V and their subcriteria (applicable to types I, II and III). Right: Patterns of calcification. (EMUC-CT = *Echinococcus multilocularis* Ulm Classification for Computed Tomography). The two columns of the classification are primarily considered separately and can then in principal be freely combined. There are two exceptions: The pattern of calcification “with a central calcification*” can only occur with primary morphology type IV* and primary morphology type V is not further characterised by a pattern of calcification. Lesions of types I and II with cystoid portions as well as types III and IV were evaluated (marked with a green frame).

The MRI classification developed by Kodama et al. has been available since 2003 [21] and describes five different types of AE lesions, including two patterns of the cystic component: small round cysts and large and/or irregular cysts. The *Echinococcus multilocularis* Ulm Classification for Ultrasound (EMUC-US) was introduced by Kratzer et al. in 2015. This classification also enables the differentiation of five different morphological types of hepatic AE lesions [23], including pseudocystic and metastatic-like forms. The *Echinococcus multilocularis* Ulm classification for computed tomography (EMUC-CT) introduced by Graeter et al. in 2016 [12] offers the basis for the systematic description of the CT morphology of AE lesions of the liver. Five primary morphological types can be distinguished. Type I: diffusely infiltrating; II: primarily circumscribed tumour-like; III: primarily cystoid (IIIa – intermediate or IIIb – widespread, depending on extent); IV: small cystoid/metastatic; V: mainly calcified. Subcriteria with respect to the presence of cystoid portions apply to types I and II, while subcriteria for the primarily cystoid type III concern the presence of a more solid margin. In addition, 1 of 6 separately defined calcification patterns is allocated to each primary morphological type, with the exception of type V, to allow for comprehensive description of the lesion. These patterns are: no calcifications, feathery calcifications, focal calcifications, a central calcification, diffuse calcifications and calcifications primarily at the edge. The pattern “with a central calcification” is a feature of type IV only (Fig. 1).

Alveolar echinococcosis (AE) has to be distinguished from cystic echinococcosis (CE), caused by the larvae of the dog tapeworm (*Echinococcus granulosus*). CE is the most common zoonosis [8] but follows a significantly more benign course than AE. CE involves mainly the liver and lungs [27].

Hepatic cysts are the most common incidental finding among focal liver lesions seen on contrast-enhanced CT [36]. Simple cysts are congenital in origin, developing from abnormal biliary tract cells and contain clear, sometimes yellowish to bile-coloured fluid [35]. They appear on CT scans as sharply demarcated hypodense lesions with a density of 0–30 Hounsfield Units (HU), i.e. isodense with fluid [29, 30]. After the administration of contrast medium, they show no enhancement and no relevant change in density (>10 HU). If there has been bleeding into the cyst or the cyst contains protein, the density may already be more than 30 HU in the non-contrast scan [29]. Cysts are therefore important in the differential diagnosis of AE lesions. Any cystic hepatic lesion has to be assessed for further benign and malignant changes. Possible lesions include non-neoplastic lesions such as liver abscesses, CE cysts, Caroli disease, bilomas, as well as neoplastic lesions such as cystadenomas, cystadenocarcinomas, or necrosis in hepatocellular carcinomas (HCC) or metastases [21, 24, 30–32].

The present study compares the density of simple hepatic cysts with the density of cystoid portions of various EMUC-CT types on the basis of HU measurements. This should further characterise the various primary morphological types of AE, and in some cases could help to make a differential diagnostic distinction especially between particular AE lesions with a primarily cystoid aspect, such as type III without more solid portions at the edge as well as type IV, and simple hepatic cysts.

## Materials and methods

### Study design

Ulm University hospital administers a national database for alveolar echinococcosis. From this database, we retrospectively extracted data for patients who had an 18F-FDG PET/CT scan in the Department of Nuclear Medicine between May 2005 and December 2017. All cases had confirmed or probable AE according to the WHO case definition but had not undergone surgery on the liver by the time of the initial PET/CT examination. We classified the hepatic lesions using the EMUC-CT. Cases that were classified as type I or type II without cystoid portions or as the predominantly calcified type V were excluded from the study.

The study population included patients who were treatment-naïve at the time of the examination, as well as those who had already started treatment with BMZ derivatives: albendazole (ABZ) or mebendazole (MBZ). This resulted in a study population of *n* = 155 subjects.

The control group consisted of patients without AE or CE, who had an 18F-FDG PET/CT scan performed between October 2014 and December 2017 for other reasons (e.g. cancer screening, staging of a known primary tumour, looking for a focus of infection), in whom hepatic cysts had been detected. The control group with simple cysts consisted of *n* = 78 patients. The whole study population therefore consisted of *n* = 233 patients.

Patient data collected from both groups included sex and age. Data were extracted retrospectively from the digital medical records of the database system (Systems, Applications and Products in Data Processing [SAP]) of the Ulm University Hospital according to the medical letter drafted at the time of the examination.

Examinations were carried out with two different PET/CT scanners: Discovery LS from General Electric (CT: Lightspeed plus; collimation 4 × 5.0 mm) and Biograph mCT-S(40) from Siemens (CT: Somatom Definition AS 40; collimation 16 × 1.2 mm). The CT data from each PET/CT examination were used to evaluate density. The portal venous phase (*n* = 135; 87.1%) and the non-contrast dataset (*n* = 20; 12.9%) were both evaluated for the AE patients; only the contrast-enhanced scans were used for the control group. Images obtained elsewhere were not taken into consideration.

**Figure 2:**
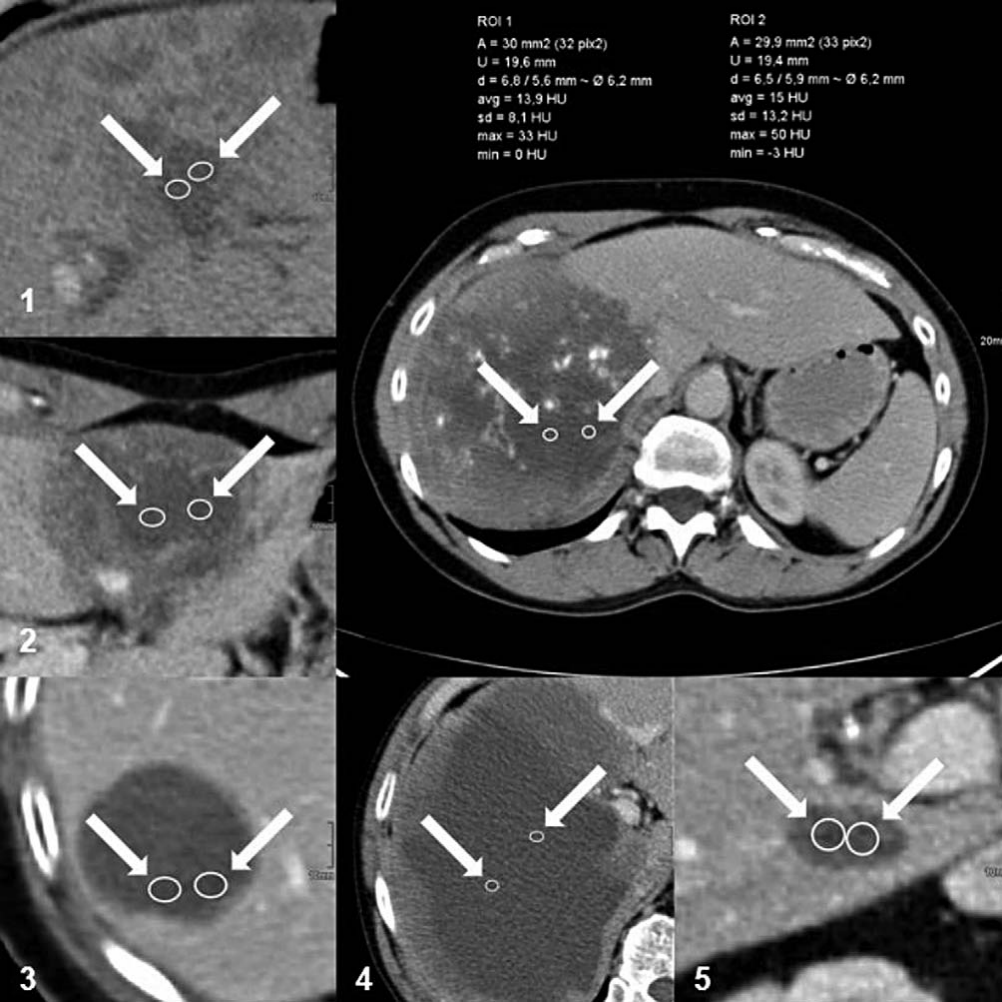
Example of density measurement in the cystoid portions of AE lesions on CT. Left and below: in each case, two enlarged ROIs within the cystoid portions of AE lesions of different primary morphological types: (1) Type I “diffuse infiltrating with cystoid portion”; (2) Type II “primarily circumscribed tumour-like with cystoid portion”; (3) Type IIIa “primarily cystoid intermediate without solid portion at the edge”; (4) Type IIIb “primarily cystoid widespread with solid portion at the edge”; (5) Type IV “small cystoid/metastatic”. Above right: two ROIs with surface data and HU values (max, min, mean and standard deviation) within the cystoid portion of an AE lesion involving both lobes of the liver. (AE = alveolar echinococcosis; CT = computed tomography; HU = Hounsfield unit; ROI = region of interest).

Measurements in the patients with AE were performed twice, by two specialty trainee radiologists with different experience (about 3 years and 6 years, respectively). They performed independent assessments from the multiplanar reconstructions (MPRs) with 4 mm or 5 mm slices using the Picture Archiving and Communication System (PACS) programme. Measurements in the control group were carried out once only (by the specialty trainee with about 6 years’ experience). For both groups, the largest cyst or the largest hepatic AE lesion with a cystoid portion was selected as the reference lesion. Its location in the lobe and segment of the liver, its largest diameter and the density of the cyst or cystoid portion of the lesion were recorded. For this purpose, the examiner selected a transverse slice with the lowest optical density in the area of the selected lesion and made two separate measurements in manually outlined round or elliptical regions of interest (ROIs) with an area of 30 ± 1 mm^2^ (Fig. 2). The density was measured in HU, recording the minimum and maximum values and calculating the mean and standard deviation (*SD*). Data were collected in tabular form using Microsoft Excel.

### Ethics statement

The study was carried out in accordance with the Declaration of Helsinki. The study protocol was approved by an independent local ethics committee (Application no 409/15). As the study was retrospective, it was not possible to obtain written informed consent from the patients. For this reason, all data were anonymised prior to the statistical analysis.

### Statistical analysis

We used SAS Version 9.4 software (SAS Institute Inc., Cary, NC, USA) for the statistical analysis of the data. The Shapiro–Wilk test was used to check the normal distribution of the data. Differences between two independent groups were tested with the Wilcoxon-Mann–Whitney test. A *p*-value of 5% (<0.05) was considered statistically significant and was reported to four decimal places. Correlation between selected values was assessed using the Spearman’s rank correlation coefficient (*r*_Sp_).

## Results

### Sex and age in the study and control groups

The study group of patients with AE comprised 90 women (58.0%) and 65 men (42.0%) aged between 18 and 87 years, with a mean age of 54.5 ± 17.4 years at the time of the examination. The control group of patients with hepatic cysts consisted of 46 women (59.0%) and 32 men (41.0%) aged between 38 and 76 years. The mean age was 62.2 ± 8.7 years.

### Lesion size in the study and control groups

Lesions in patients with AE were between 8 and 210 mm in size, with a mean of 79.3 ± 48.1 mm. The cysts were significantly smaller with a diameter between 13 and 100 mm and a mean of 30.9 ± 16.6 mm (*p* < 0.0001) (Table 1).

**Table 1:** Patient characteristics in the study group (*n* = 155) and the control group (*n* = 78).

Characteristic	Study group	Control group
	(*n* = 155)	(*n* = 78)
	*N* (%)	
Sex		
Male	65 (42.0)	32 (41.0)
Female	90 (58.0)	46 (59.0)
Age at time of examination		
<18 years	0 (0.0)	0 (0.0)
18–50 years	61 (39.3)	8 (10.2)
51–60 years	30 (19.3)	26 (33.3)
61–70 years	27 (17.4)	28 (35.9)
>70 years	37 (23.8)	16 (20.5)
Pharmacotherapy		
No treatment	49 (31.6)	
≤35 days	21 (13.5)	
>35 days	65 (41.9)	
Treatment and interruption	20 (12.9)	
	Mean ± *SD*	
	Min – Max	
HU value (unit)	21.8 ± 17.6	2.9 ± 4.5
	−16.4 – 76.9	−9.9 – 10.8
Age at examination (years)	54.5 ± 17.4	62.2 ± 8.7
	18 – 87	38 – 76
Size of the space-occupying lesion (mm)	79.3 ± 48.1	30.9 ± 16.6
	8 – 210	13 – 100

HU = Hounsfield unit; *SD* = standard deviation; max = maximum; min = minimum.

### Distribution of the lesions in the lobes of the liver in the study and control groups

In the study group, the largest AE lesions were found in the right lobe of the liver in 86 cases (55.4%) and in the left lobe in 36 patients (23.2%). In 33 cases (21.2%), the AE lesions traversed the border between the lobes and were therefore present in both lobes. Figures for the simple cysts were 34 (43.5%) in the right lobe and 44 (56.4%) in the left lobe. None of the cysts extended into both lobes. The difference in distribution of the lesions between both groups was statistically significant (*p* < 0.0001).

### Primary morphology of the AE lesions

The frequency of the primary morphology types in the 155 AE lesions was type I 25.8% (*n* = 40), type II 14.8% (*n* = 23), type III 31.6% (*n* = 49) (of which 16.7% [*n* = 26] were type IIIa and 14.8% [*n* = 23] were type IIIb) and type IV 27.7% (*n* = 43). The type III lesions were subdivided: 63.2% (*n* = 31) with a solid portion at the edge and 36.7% (*n* = 18) without a solid portion at the edge. Type IIIa consisted of 46.2% (*n* = 12) with solid portion at the edge and 53.8% (*n* = 14) without a solid portion at the edge, while type IIIb consisted of 82.6% (*n* = 19) with a solid portion at the edge and 17.4% (*n* = 4) without a solid portion at the edge.

### Calcification pattern of the AE lesions

The calcification pattern of the lesions was as follows: no calcifications *n* = 40 (25.8%), feathery calcifications *n* = 19 (12.2%), diffuse calcifications *n* = 36 (23.2%), focal calcifications *n* = 21 (13.5%), calcifications primarily at the edge *n* = 23 (14.8%), and a central calcification *n* = 16 (10.3%).

### HU values in the study and control groups

The inter-rater reliability for the measurement of the AE lesions was 92%.

**Figure 3:**
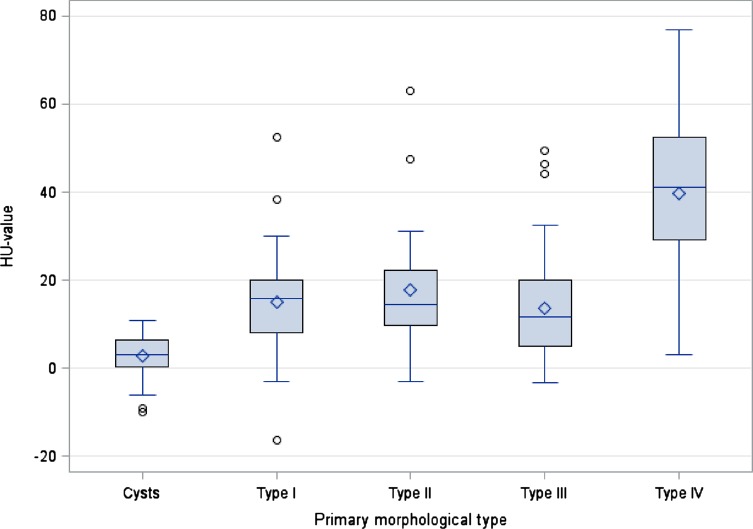
Box plot showing the HU values and spread of the EMUC-CT primary morphological types and cysts.

Density measurements of the cystoid portions of each primary morphological type gave the following means: type I 14.9 ± 11.5 HU, type II 17.7 ± 14.2 HU, type III 13.5 ± 11.5 HU (type IIIa 16.5 ± 13.8 HU and type IIIb 10.3 ± 7.0 HU). Type IV showed by far the highest density with 39.8 ± 17.2 HU; this was significantly different from the other types (I, II and III) (*p* < 0.0001) (Table 2). Density measurements of all the cystoid portions of the AE lesions (types I–IV) together gave a mean of 21.8 ± 17.6 HU with a spread between −16.4 and 76.9 HU. This was significantly different (*p* < 0.0001) from the simple hepatic cysts, which showed a much lower density at a mean of 2.9 ± 4.5 HU (min – max −9.9 – 10.8 HU). The cystoid portions of the individual AE types (I–IV) also differed significantly in density from the simple cysts (*p* < 0.0001) in each case (Table 3). In addition, there were significant differences in the HU values of the cystoid portions in types I, II and IIIa/b and the simple cysts compared with type IV (*p* < 0.0001) (Fig. 3, Table 3).

**Table 2:** HU values in the study group (*n* = 155) according to primary morphological type and in the control group (*n* = 78).

Morphology	*N*	Mean HU value ± *SD*	Min – Max HU value	*p*-value
Type I	*n* = 40	14.9 ± 11.5	−16.4–52.5	*p* < 0.0001
Type II	*n* = 23	17.7 ± 14.2	−3.0–63.1	
Type III	*n* = 49	13.5 ± 11.5	−3.4–49.5	
Type IIIa	*n* = 26	16.5 ± 13.8	−3.4–49.5	
Type IIIb	*n* = 23	10.3 ± 7.0	−3.3–24.3	
Type IV	*n* = 43	39.8 ± 17.2	3.0–76.9	
Cysts	*n* = 78	2.9 ± 4.5	−9.9–10.8	

HU = Hounsfield unit; *SD* = standard deviation; max = maximum; min = minimum. *p* < 0.05 statistically significant.

**Table 3:** Difference in the HU values of primary morphological types I–IV compared with cysts, as well as of primary morphological types I–III and cysts compared with type IV.

Morphology	Mean HU value ± *SD*					
	Min – Max HU value					
	Type X	Cysts	*p*-value	Type X	Type IV	*p*-value
Type I	14.9 ± 11.5	2.9 ± 4.5	<0.0001	14.9 ± 11.5	39.8 ± 17.2	<0.0001
	−16.4–52.5	−9.9–10.8		−16.4–52.5	3.0–76.9	
Type II	17.7 ± 14.2	2.9 ± 4.5	<0.0001	17.7 ± 14.2	39.8 ± 17.2	<0.0001
	−3.0–63.1	−9.9–10.8		−3.0–63.1	3.0–76.9	
Type III	13.5 ± 11.5	2.9 ± 4.5	<0.0001	13.5 ± 11.5	39.8 ± 17.2	<0.0001
	−3.4–49.5	−9.9–10.8		−3.4–49.5	3.0–76.9	
Type IV/cysts	39.8 ± 17.2	2.9 ± 4.5	<0.0001	2.9 ± 4.5	39.8 ± 17.2	<0.0001
	3.0–76.9	−9.9–10.8		−9.9–10.8	3.0–76.9	

HU = Hounsfield Unit; *SD* = standard deviation; max = maximum; min = minimum. *p* < 0.05 statistically significant.

### Correlations within the study group

Density measurements of the cystoid portions of AE lesions showed a correlation between the pattern of the primary morphology and the HU value (*r* = 0.43139). Comparison of the size of the lesion with the density demonstrated a high correlation between the two parameters (*r* = −0.60837). The smaller the lesion, the higher the HU value. Both of these comparisons were statistically significant (*p* < 0.0001). In contrast, comparison of the underlying calcification patterns and the density of the cystoid portions gave a low correlation coefficient (*r* = −0.04004) and was not significant (*p* < 0.6220).

## Discussion

Imaging plays a key role in the diagnostic investigation of AE [1, 5, 28]. As earlier studies have shown, AE lesions show a broad spectrum of morphological characteristics on ultrasound, CT and MRI allowing various types to be distinguished. One criterion is the possible presence of liquid components of the lesions. The EMUC-US [23] defined a pseudocystic pattern, and the MRI classification from Kodama et al. [21] describes small round cysts as well as large and/or irregular cysts. Nevertheless, the classical picture of a cyst in the strictest sense is not seen in AE but is possibly reflected as “small cysts” in the sense of alveoli. The EMUC-CT has established the term “cystoid” for the confluent hypodense areas of the lesions.

When cyst-like lesions or parts of lesions occur in AE, their appearance in the different imaging modalities may present a real challenge in the differential diagnostic distinction from simple cysts, the most common focal liver lesions [17, 24, 36]. Distinguishing these two entities from hepatic *Echinococcus granulosus* infestations is a further challenge [27, 38]. It must be remembered that the different types of CE lesion may sometimes closely resemble simple cysts, depending on the stage of the disease [28]. The serological evidence that contributes to the diagnosis may be conflicting in both parasitic infestations. It may therefore delay an early diagnosis or even foster a misdiagnosis. Positive serology alone is not sufficient to confirm the diagnosis of echinococcosis [39].

The CT modality offers the possibility of determining the density of structures quantitatively by measuring HU values [15]. Already in 1985, Didier et al. reported CT findings in a small population of 24 patients with hepatic AE lesions showing on the one hand heterogeneous hypodense areas without contrast medium enhancement with densities of 20–40 HU in 92% of cases, and on the other hand pseudocystic areas with densities of 0–10 HU within the hypodense regions with a “geographical map” pattern in 40% of cases [9]. Liu et al. found that some patients with AE had cystic cavities with densities between 3 and 10 HU on CT after ABZ therapy, concluding that these cavities contained serous fluid [26]. Reuter et al. too reported CT findings of central hypodense necrotic areas and densities of 0–25 HU in AE lesions [31]. The present study is the first to compare simple hepatic cysts with the cystoid portions of various EMUC-CT types of AE lesion on the basis of the HU value in a large patient population (*n* = 155).

The ratio of female to male subjects in the study group with AE (58.0% women and 42.0% men) reflects previous observations that women are more commonly affected [13, 14, 16, 19, 20, 22, 31, 37]. Nevertheless, it has to be remembered that our exclusion criteria (AE primary morphology type I and type II without cystoid portions, type V, patients who had undergone surgery and patients whose diagnostic imaging had been performed elsewhere) meant that not all the patients in the local *Echinococcus* database were included in the study. The presence of more female patients in the control group agrees with the prevalence of hepatic cysts found in other studies to date, although these were not always statistically significant [11, 17, 25, 35].

With respect to the density of the cystoid portions of the hepatic AE lesions overall compared with the density of simple hepatic cysts, our study showed that the cystoid components in AE have significantly higher HU values than hepatic cysts (21.8 ± 17.6 HU vs 2.9 ± 4.5 HU). This difference was also significant when comparing the simple hepatic cysts with each of the individual AE types. This result is clinically relevant, as a distinction between the two entities may be crucial in the context of differential diagnosis, especially with regard to the primarily cystoid AE types III and IV. Furthermore, if cysts occur polycyclically or partially septated, they can assume very bizarre formations in CT, so that the differentiation from the other manifestations of AE must also be made. The HU value measurement can be one mosaic stone in the first approach to differential diagnosis in this context. The present results also emphasise the significance of the terminology selected for the EMUC-CT. Types I and II of the five primary morphological lesions may contain hypodense regions as a subcriterion, while such areas are already included in the primary description of types III and IV, being correctly designated “cystoid” rather than “cystic”.

The cystoid portions in diffuse infiltrating type I had a mean density of 14.9 ± 11.5 HU and in primarily circumscribed tumour-like type II a mean density of 17.7 ± 14.2 HU. In relation to the whole study population, these types had a moderately high increase in density. It is possible that the cystoid portions in these two groups either still consisted of alveolar conglomerations with intervening septa or of areas of still denser necrosis with incipient liquefaction. Septa included in the measurements or areas of necrosis undergoing initial liquefaction but still relatively protein rich could lead to an increased density of this nature. The mean HU value for type III (type IIIa/b together) was 13.5 ± 11.5 HU, with values tending to be lower than those of types I and II. Type IIIb, which is primarily cystoid with extensive lesions, notably had the lowest density of any AE type at 10.3 ± 7.0 HU and was therefore closest to the hepatic cysts. This can be explained by the fact that the cystoid portions here comprised a single area several centimetres across, consistent with necrosis in an advanced stage of liquefaction, as was discussed when the classification was established in 2016 [12]. Evidence that type III is actually older AE lesions has been supplied by a recent international comparative study [13]. The relationship that we observed between the size of the lesion and the density of the cystoid portions shows a high correlation (*r* = −0.60837) and we can conclude: the smaller the lesion, the higher the HU value. This would also support the hypothesis outlined above.

**Figure 4:**
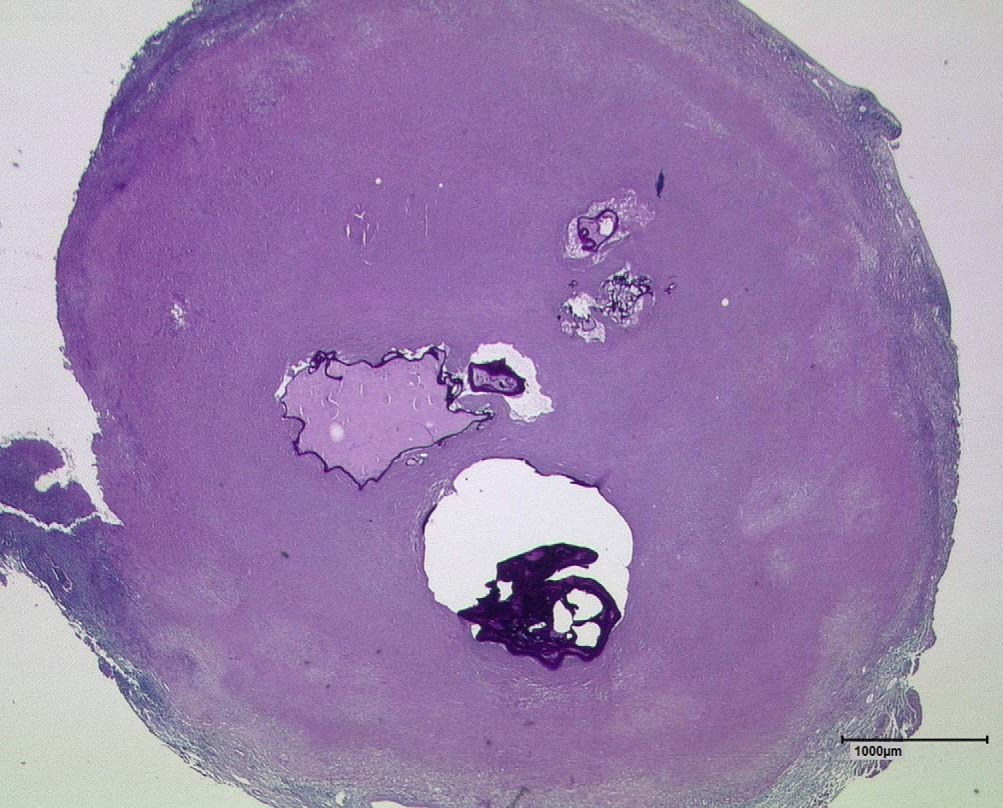
Section of a type IV AE lesion with central alveoli after PAS staining at 12.5× magnification. Note the wide surrounding area of solid necrosis. (AE = alveolar echinococcosis, PAS = periodic acid-Schiff).

Regarding the correlation between lesion size and density, the very small type IV consequently has an exceptional position in the five EMUC-CT primary morphology types. This type of lesion showed by far the highest density, with a mean of 39.8 ± 17.2 HU. Type IV was therefore significantly different not only from simple cysts but also from the cystoid portions of types I, II and III. Type IV lesions usually consist of one small alveolus (sometimes even a few) surrounded by solid necrosis of varying width (Fig. 4). In the CT images, this wide band of necrosis also appears hypodense, although to a lesser extent than the alveoli. Given a sufficiently pronounced central alveolus, these lesions may therefore also furnish sensitive evidence in T2-weighted MRI [2]. The existing dichotomy in the structure of type IV lesions leads to higher density values than found in the other types: density measurements within the ROI give lower HU values if an alveolus of a certain size is present, whereas they increase if the alveolus is very small or even absent. The two main lesion characteristics of type IV are described as small cystoid/metastatic according to the previous EMUC-CT terminology. As the term is intended to describe only the morphological appearance and not any metastatic potential of these lesions, we propose that in future it should be “small cystoid/metastasis-like”, which is less likely to be misunderstood.

Finally, the aspect of the different densities within the cystoid portions of the various types that we have discussed above, fits well with the evolutionary hypothesis of AE lesions outlined in a recent multicentre study [13]. In this context, there are intermodal parallels to important observations made with Kodama’s MRI classification [3, 21]. It would be interesting for future studies to compare CT and MRI findings, as current knowledge suggests parallels between the classification of the cystoid portions in EMUC-CT types I, II and III and types 1, 3 and 5 of Kodama’s classification. Such a study could also demonstrate that the Kodama classification does not yet have an equivalent to the small initial, potentially either intensifying or involuting, type IV of the EMUC-CT.

One limitation of the study was the significant age difference between the study group and the control group. This difference may be explained firstly by the fact that the prevalence of hepatic cysts increases with age, and cysts in patients below the age of 40 are a rarity [7, 11, 17, 25, 35]. Secondly, the PET/CT scans were frequently carried out in the control group because of cancer; this patient group is also one of more advanced age. Furthermore the study does not provide a juxtaposition of CT morphology and histopathology.

## Conclusions

Depending on the morphology, cystoid components may be found in hepatic AE lesions. For this reason, particular AE lesions belong, besides initial stages of CE, to the most important conditions in the differential diagnosis of the most common focal liver lesions, benign hepatic cysts. With significantly higher values, density measurements of cystoid hepatic AE lesions offer a good opportunity for distinguishing them from simple hepatic cysts. There is a relationship between lesion size and density value, possibly as the expression of lesions in different stages of advancement. The highest density of type IV, which is significantly different from all the other types of lesions, fits the structure of this lesion which, in addition to hypodense solid necrosis may also have a central alveolus varying in size as a sign of vitality. The results also emphasise the significance of the terminology selected for the EMUC-CT, using the term “cystoid” rather than “cystic”.
